# Barriers and facilitators to exercise in hemodialysis patients: A protocol for systematic review

**DOI:** 10.1097/MD.0000000000023129

**Published:** 2020-12-11

**Authors:** Tianzi Li, Aili Lv, Na Xu, Mei Huang, Yan Su

**Affiliations:** a Health Science Center, Xi’an Jiaotong University; b The Fourth Military Medical University, Xi’an, Shaan Xi, China.

**Keywords:** exercise, hemodialysis, systematic review

## Abstract

**Background:**

Exercise training has a lot of potential benefits for hemodialysis patients. And some guidelines emphasize the importance of exercise for maintenance hemodialysis. However, there are many barriers to encourage hemodialysis patients to increase their level of physical activity. A broader understanding of the specific barriers is needed.

**Methods:**

MEDLINE, EMBASE, Web of Science, CINAHL, PsycINFO, PubMed, CBM, CNKI, and WangFang will be searched electronically. The Reference lists of included studies will be retrieved manually. If the study is designed with qualitative or mixed methods and directly explores the factors related to the exercise of dialysis patients, the study will be selected. The Critical Appraisal Skills Programme Qualitative Checklist will be applied for the study appraisal. The study search, selection and evaluation of the study will be conducted by 2 independent reviewers. Thematic synthesis will be used for synthesizing the findings of the primary studies.

**Results:**

This study will provide a high-quality synthesis to examine the barriers and facilitators affecting exercise in hemodialysis patients from the perspective of patients, caregivers, and health care providers.

**Conclusion:**

This systematic review will contribute to the in-depth understanding of barriers and facilitators affecting exercise in hemodialysis patients, and improve the prognosis of this population.

**Ethic and dissemination:**

The content of this article does not involve moral approval or ethical review because no individual data will be collected.

**PROSPERO registration:**

CRD42020200278. (https://www.crd.york.ac.uk/prospero/#recordDetails).

## 1 Introduction

The number of patients with chronic kidney failure, which is increasing by 5% to 6% a year, is one of the most important problems in every country, and hemodialysis is the main treatment for chronic kidney failure.^[1]^ Dialysis leads to distinct metabolic changes such as hypovolemia, electrolyte imbalance, and systemic inflammation.^[2]^ In addition, patients suffer from protein energy expenditure, muscle wasting, and depression, which also limit their physical activity.^[3,4]^ As a result, the exercise capacity in hemodialysis patients is about 50% to 60% of that seen in normal subjects.^[5]^ Given the fact that low physical activity is frequent and related to morbidity and mortality, the Kidney Disease Outcomes Quality Initiative Clinical Practice Guidelines recommend to routinely counsel hemodialysis patients on increasing their physical activity levels.^[6,7]^ Exercise training has a lot of potential benefits. Chronic regular exercise can improve the aerobic capacity, muscle strength, and cardiovascular function of dialysis patients.^[8]^ In addition, exercise was highly positively correlated with dialysis efficacy, high-sensitivity C-reactive protein, blood pressure, arterial stiffness, health-related quality of life, and depression.^[8,9]^

And some guidelines emphasize the importance of exercise for maintenance hemodialysis. However, there are many barriers to encourage hemodialysis patients to increase their level of physical activity.^[10]^ A broader understanding of the specific barriers is needed. These data may be highly relevant to changes in studies that improve exercise levels and survival outcomes in hemodialysis patients. So far, no systematic studies have identified these factors. Qualitative research, which explores human experience in depth, may be better suited to elucidate the barriers and facilitators of exercise participation in hemodialysis patients.

The main objective of this review is to systematically review qualitative literature related to exercise in hemodialysis patients, with a particular focus on the barriers and facilitators affecting exercise in hemodialysis patients from the perspective of patients, caregivers and health care providers (HCPs).

## 2 Methods

This systematic review protocol follows the Preferred Reporting Items for Systematic Review and Meta-analysis Protocols (PRISMA-P) 2015 statement.^[11]^ The systematic review was registered with the International Prospective Register of Systematic Reviews (PROSPERO), registration number CRD42020200278(https://www.crd.york.ac.uk/prospero/#recordDetails).

### 2.1 Inclusion criteria

#### 2.1.1 Types of studies

Types of studies were qualitative studies and mixed methods studies which express the opinion about barriers and facilitators of exercise in hemodialysis patients.

#### 2.1.2 Types of participants

1. Hemodialysis patients older than 18 years with at least 3 months of hemodialysis and normal cognitive function are included.
2. Caregivers who live with hemodialysis patients and provide emotional support and assistance in their daily lives are included.
3. Registered HCPs working with patients in hemodialysis are included.

#### 2.1.3 Types of outcomes

Barriers and facilitators affecting exercise of hemodialysis patients reported by hemodialysis patients, caregivers and HCPs.

#### 2.1.4 Language

Studies will be included if written in English and Chinese.

### 2.2 Search strategy

The search strategy will comprise comprehensive keyword combinations for each of the three concepts of interest, that is, hemodialysis, exercise, qualitative study design.

We will search English databases including MEDLINE (from 1950 to July 2020), EMBASE (from 1974 to July 2020), Web of Science (from 1900 to July 2020), CINAHL (from 1981 to July 2020), PsycINFO (from 1806 to July 2020), and PubMed (from 1950 to July 2020). Chinese databases include CBM (from 1978 to July 2020), CNKI (from 1979 to July 2020), and WangFang (from 1900 to July 2020). In addition to the mentioned search strategy, we will manually search reference lists of included studies to identify any additional studies that fit the inclusion criteria. We detail the electronic search strategy in Table 1, using PubMed as an example.

**Table 1:** Search strategy to be used for the PubMed electronic database.

Database	Search terms
PubMed	#1 ((“Intradialytic”[All Fields] OR (“hemodialysis”[All Fields] OR “renal dialysis”[MeSH Terms] OR (“renal”[All Fields] AND “dialysis”[All Fields]) OR “renal dialysis”[All Fields] OR “hemodialysis”[All Fields]) OR (“hemodialysis”[All Fields] OR “renal dialysis”[MeSH Terms] OR (“renal”[All Fields] AND “dialysis”[All Fields]) OR “renal dialysis”[All Fields] OR “hemodialysis”[All Fields]) OR (“renal dialysis”[MeSH Terms] OR (“renal”[All Fields] AND “dialysis”[All Fields]) OR “renal dialysis”[All Fields]) OR (“renal dialysis”[MeSH Terms] OR (“renal”[All Fields] AND “dialysis”[All Fields]) OR “renal dialysis”[All Fields] OR (“extracorporeal”[All Fields] AND “dialyses”[All Fields]) OR “extracorporeal dialyses”[All Fields]) OR (“renal dialysis”[MeSH Terms] OR (“renal”[All Fields] AND “dialysis”[All Fields]) OR “renal dilysis”[All Fields] OR (“extracorporeal”[All Fields] AND “dialysis”[All Fields]) OR “extracorporeal dialysis”[All Fields]) OR (“renal dialysis”[MeSH Terms] OR (“renal”[All Fields] AND “dialysis”[All Fields]) OR “renal dialysis”[All Fields] OR “dialysis”[All Fields] OR “dialysis”[MeSH Terms]) OR (“dialysis”[MeSH Terms] OR “dialysis”[All Fields] OR “dialyses”[All Fields]))
	#2 ((“exercise”[MeSH Terms] OR “exercise”[All Fields]) OR (“intradialytic”[All Fields] AND (“exercise”[MeSH Terms] OR “exercise”[All Fields])) OR (“physical therapy”[MeSH Terms] OR (“physical”[All Fields] AND “therapy”[All Fields]) OR “physical training” All Fields]) OR (“physical training”[MeSH Terms] OR (“physical”[All Fields] AND “training”[All Fields]) OR “physical activity”[All Fields] OR (“physical”[All Fields] AND “activity”[All Fields]))
	#3 ((“qualitative study”[Publication Type] OR “qualitative study as topic”[MeSH Terms] OR “qualitative study”[All Fields]) OR (“qualitative research”[MeSH Terms] OR (“qualitative research”[All Fields]) OR (“qualitative description”[MeSH Terms] OR “qualitative description”[All Fields]) OR (“phenomenological study”[MeSH Terms] OR “ phenomenological study”[All Fields]) OR (“grounded theory”[MeSH Terms] OR “grounded theory”[All Fields]) OR (“interview”[MeSH Terms] OR “interview”[All Fields]))
	#4 #1 AND #2 AND #3

### 2.3 Study selection

**Figure 1:**
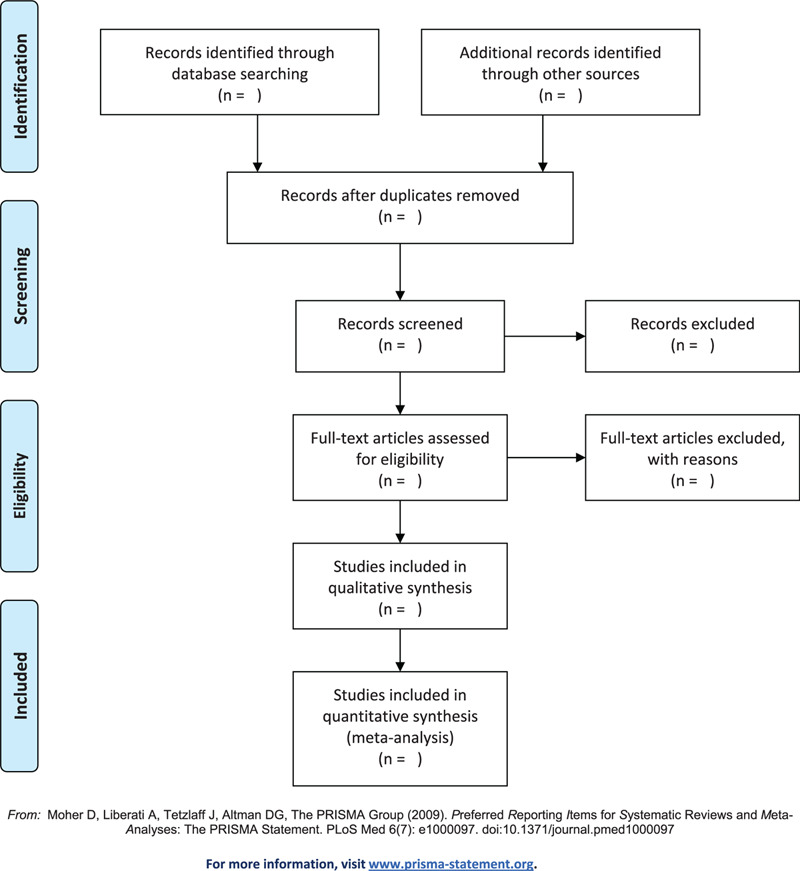
Flow chart of the study selection.

The search results will be imported into the Endnote X9 software. Duplicate and unrelated studies will be deleted. Two independent evaluators will first screen the study title and abstract to determine its substitutability. Eligibility for each study will be tested against predefined eligibility criteria and quality assessment guidelines. In all cases, the decision to include or exclude a study must be approved by 2 reviewers. If a decision cannot be made, a third reviewer will make the final decision. A flow chart using PRISMA's reporting guidelines will be used to report the selection process and results (Fig. 1).

### 2.4 Appraisal of study quality

Critically Appraisal Skills Programme (CASP) checklist^[12]^ in Table 2 was used to assess the quality of studies. CASP evaluates qualitative research mainly from the rigor of the design, the credibility of the results, and the relevance of the results to existing practice. The fully satisfied standard is Level A, partially satisfied standard is level B, and completely unsatisfied standard is level C. Studies at level A and B were eventually included. Each study must meet the minimum objective criteria, if not, the study will be deleted.

**Table 2:** Critically appraisal skills programme checklist.

Question	Yes	Unclear	No
1. Is this study qualitative research?			
2. Are the research questions clearly stated?			
3. Have ethical issues been taken into consideration?			
4. Is the qualitative approach clearly justified?			
5. Is the approach appropriate for the research question?			
6. Is the study context clearly described?			
7. Is the role of the researcher clearly described?			
8. Is the sampling method clearly described?			
9. Is the sampling strategy appropriate for the research question?			
10. Is the method of data collection clearly described?			
11. Is the data collection method appropriate to the research question?			
12. Is the method of analysis clearly described?			
13. Is the chosen analytical approach suitable for addressing the research question?			
14. Are the claims made supported by sufficient evidence?			

The review process involves 2 authors independently reviewing each study based on a checklist to reach a consensus. If inconsistencies occur, a third examiner will determine the outcome.

### 2.5 Data collection/extraction

Two authors will independently extract data. Any disagreement will be resolved by discussion until consensus is reached by consulting a third author. Extracted data will include, but not be limited to country, sample size, methodological, data collection, analysis method, and results (including barriers and facilitators of exercise).

### 2.6 Data synthesis

The thematic synthesis described by Thomas and Harden will be applied to the data synthesis.^[13]^ Thematic synthesis consists of three stages: line by line free coding; construct descriptive themes; construct analytical themes.

We carefully read each included study, extracted and coded the topics explored in each study regarding the barriers and facilitators affecting exercise in hemodialysis patients. Then grouped similar topics and developed new codes when necessary. The comprehensive study will be conducted by one author and examined by another independent author with experience in thematic synthesis to enhance credibility. Tables and visual representations of the thematic synthesis will be provided.

### 2.7 Patient and public involvement

Since this is a study Protocol for a systematic review of qualitative studies, and no raw data will be collected from the patient, there is no need for patient and public involvement.

## 3 Discussion

To our knowledge, this will be the first study to systematically review and synthesize qualitative data on exercise barriers and facilitators in hemodialysis patients from the perspective of patients, caregivers, and HCPs. The focus on qualitative research results will provide a reference for the exercise practice of hemodialysis patients, so as to make wider use of these data. These findings will shed light on factors that impede or promote exercise in hemodialysis patients.

This protocol details the reasonableness and methodology of the proposed system review. In case any deviation from the protocol takes place, it will be justified and discussed in the systematic review on publication.

## 4 Contributors

TL and AL conceived and designed the initial study. TL, NX, and YS drafted the initial protocol. All authors contributed to the development of the selection criteria, the risk of a bias assessment strategy, and data extraction criteria. AL is the guarantor of the review. All authors read, provided feedback, and approved the final protocol before submission to the journal.

## Author contributions

**Conceptualization:** TianZi Li, Aili Lv.

**Data curation:** TianZi Li, Na Xu, Mei Huang.

**Formal analysis:** TianZi Li, Na Xu, Mei Huang, Yan Su.

**Supervision:** Aili Lv, Mei Huang.

**Writing – original draft:** TianZi Li, Yan Su.
